# Supplementary material for: Chinese Cabbage Changes Its Release of Volatiles to Defend against Spodoptera litura
Source: Insects. 2022 Jan 10;13(1):73. doi: 10.3390/insects13010073 (PMC8778687; doi:10.3390/insects13010073)
Supplement: Supplementary file 1 [file insects-13-00073-s001.zip › insects-1536714-supplementary.pdf]

Table S1 Volatiles released by Chinese cabbage plants as affected by six treatments.

Number	Retention time (S)	Compound	HP	MDP	RTP	SIP12	SIP24	SIP48
1	7.214	(E) -2-hexenal	+	+	+	+	−	−
2	7.439	(Z)-3-hexen-1-ol	+	+	+	+	+	+
3	10.872	Butane, 1-isothiocyanato	+	+	−	+	−	−
4	14.082	1-Butene, 4-isothiocyanato	+	+	+	+	+	+
5	16.424	(Z)-3-hexenyl acetate	+	+	+	+	+	+
6	17.178	Limonene	−	−	−	+	+	+
7	20.056	Allyl isothiocyanate	+	+	+	+	+	+
8	21.155	Linalool	+	+	+	+	+	+
9	21.425	Nonanal	+	+	+	+	+	+
10	25.880	Decanal	+	+	+	+	+	+
11	31.636	Benzyl isothiocyanate	+	+	+	+	+	+
12	32.948	Tetradecane	+	+	+	+	+	+
13	34.368	Geranyl acetone	+	+	+	+	+	+
14	36.086	Pentadecane	+	+	+	+	+	+
15	39.075	Hexadecane	+	+	+	+	+	+
16	41.948	Heptadecane	+	+	+	+	+	+
17	44.077	Octadecane	+	+	+	+	+	+
